# EpCAM Signaling in Oral Cancer Stem Cells: Implications for Metastasis, Tumorigenicity, and Therapeutic Strategies

**DOI:** 10.3390/cimb47020123

**Published:** 2025-02-14

**Authors:** Chuan-Hsin Chang, Chung-Che Tsai, Fu-Ming Tsai, Tin-Yi Chu, Po-Chih Hsu, Chan-Yen Kuo

**Affiliations:** 1 Department of Research, Taipei Tzu Chi Hospital, Buddhist Tzu Chi Medical Foundation, New Taipei City 231, Taiwan; chuanhsin032484@gmail.com (C.-H.C.); chungche.tsai@gmail.com (C.-C.T.); afu2215@gmail.com (F.-M.T.); tintin4125@gmail.com (T.-Y.C.); 2 Department of Dentistry, Taipei Tzu Chi Hospital, Buddhist Tzu Chi Medical Foundation, New Taipei City 231, Taiwan; 3 Institute of Oral Medicine and Materials, College of Medicine, Tzu Chi University, Hualien 970, Taiwan

**Keywords:** EpCAM, cancer, cancer stem cells, metastasis, immunotherapy, tumor microenvironment

## Abstract

Oral cancer, a subtype of head and neck cancer, poses significant global health challenges owing to its late diagnosis and high metastatic potential. The epithelial cell adhesion molecule (EpCAM), a transmembrane glycoprotein, has emerged as a critical player in cancer biology, particularly in oral cancer stem cells (CSCs). This review highlights the multifaceted roles of EPCAM in regulating oral cancer metastasis, tumorigenicity, and resistance to therapy. EpCAM influences key pathways, including Wnt/β-catenin and EGFR, modulating CSC self-renewal, epithelial-to-mesenchymal transition (EMT), and immune evasion. Moreover, EpCAM has been implicated in metabolic reprogramming, epigenetic regulation, and crosstalk with other signaling pathways. Advances in EpCAM-targeting strategies, such as monoclonal antibodies, chimeric antigen receptor (CAR) T/NK cell therapies, and aptamer-based systems hold promise for personalized cancer therapies. However, challenges remain in understanding the precise mechanism of EpCAM in CSC biology and its translation into clinical applications. This review highlights the need for further investigation into the role of EPCAM in oral CSCs and its potential as a therapeutic target to improve patient outcomes.

## 1. Introduction

**Figure 1:**
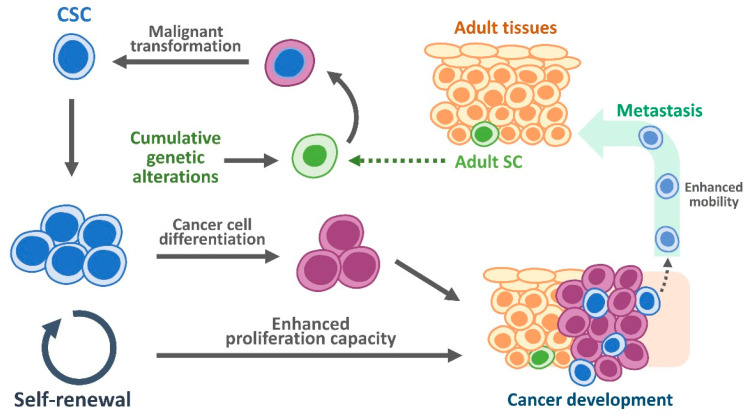
Diagram of cancer stem cells. Adult SCs can accumulate genetic alterations, such as gene mutations, which may result in their transformation into malignant cells, also known as CSCs. These CSCs are capable of self-renewing to maintain their population and differentiating into non-stem cancer cells, driving cancer development and metastasis.

Oral cancer is consistently ranked among the top ten cancers across the world, with large differences in its geographic distribution [1]. Oral cancer is a type of head and neck cancer characterized by cancerous tissue growth in the oral cavity, including the lips, tongue, cheeks, mouth floor, hard and soft palate, sinuses, and pharynx (throat). Oral cancer can be life-threatening if not diagnosed or treated early. This type of cancer can arise as a primary lesion originating from any tissue in the mouth, via metastasis from a distant site of origin, or extending from the nasal cavity. Oral cancers can originate in the mouth in various forms, such as teratoma, adenocarcinoma derived from a major or minor salivary gland, lymphoma from tonsillar or other lymphoid tissues, or melanoma from the pigment-producing cells of the oral mucosa. Studies have indicated that approximately 90% of oral cancers are oral squamous cell carcinomas (OSCC) [2,3,4], originating in the mucosal epithelium that lines the mouth and lips; however, there are other types of oral cancer, such as oral Kaposi’s sarcoma [5]. In the early stages of oral cancer, it can go unnoticed and is painless, with subtle physical changes. Treatment is generally effective if oral cancer is diagnosed at an early stage. Late (advanced) stage (stage III/IV) symptoms include indurated areas, paresthesia or dysesthesia of the tongue or lips, airway obstruction, chronic serous otitis media, otalgia, trismus, dysphagia, cervical lymphadenopathy, persistent or referred pain, and altered vision. The 5-year disease-free survival rate for intraoral carcinoma is 76% if metastasis has not occurred, 41% when the cervical nodes are involved, and only 9% when metastasis below the clavicle is present [6]. Additionally, tumor metastasis and subsequent recurrence have a negative impact on the 5-year survival rate under current therapies [7]. Cancer stem cells (CSCs) possess self-renewal and differentiation capacities that cause heterogeneous lineages of cancer cells to form tumors [8] (Figure 1). Also, it has been reported that cancer stem cells (CSCs) play a crucial role in the recurrence, metastasis, and poor prognosis in OSCC [9,10,11,12] (Figure 1).

## 2. EpCAM as a CSC Marker: Signaling Pathways and Role in Oral CSCs

**Figure 2:**
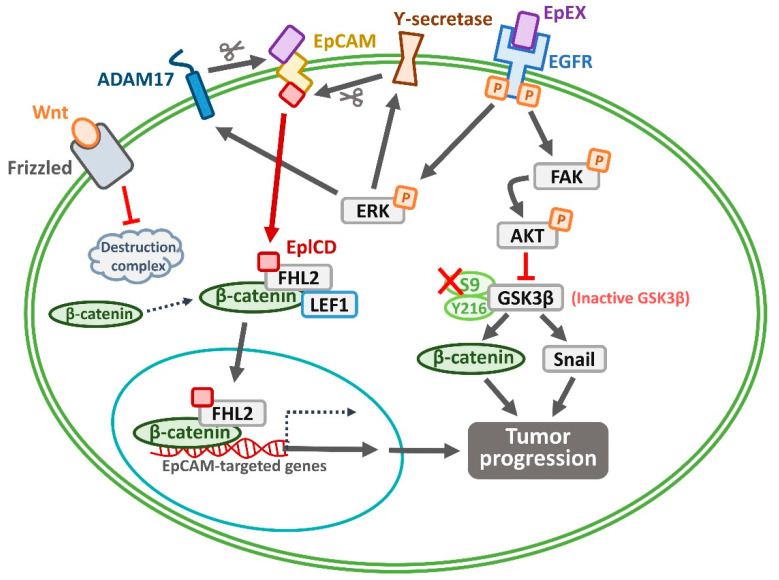
Role of the EpCAM signaling pathway in cancer progression. EpCAM undergoes sequential proteolytic processing on CSC membranes. ADAM17 and γ-secretase mediate this cleavage, releasing the extracellular domain (EpEX) and intracellular domain (EpICD). While the proteasome degrades most EpICD, a portion translocates to the nucleus where it forms a transcriptional complex with FHL2, LEF1, and β-catenin. The EpEX contains an EGF-like region that interacts with EGFR, triggering EGFR-ERK pathway activation. Concurrent activation of Wnt-β-catenin signaling leads to cytoplasmic β-catenin accumulation. FHL2 facilitates the nuclear translocation of both EpICD and β-catenin, where they form a transcriptional complex that regulates EpCAM target genes. Through these molecular mechanisms, EpCAM emerges as a central regulator of CSC in tumor progression such as proliferation, metastatic potential, and chemoresistance.

The epithelial cell adhesion molecule (EpCAM), also known as CD326 and epithelial-specific antigen (ESA), was initially proposed to function as a cell–cell adhesion molecule [13] and is a type I transmembrane glycoprotein expressed in various epithelial tissues [14,15,16]. Clinical studies have shown that EpCAM is highly expressed in various cancers, including breast cancer, pancreatic cancer [17], various epithelial cancers, and OSCC, and its overexpression is associated with poor prognosis [18,19,20,21,22,23]. Al-Hajj et al. reported that the frequency of tumor-initiating cells was >10-fold higher in the EpCAM-positive fraction of breast CSCs than in the EpCAM-negative fraction [24]. EpCAM is also a CSC marker in various cancers, including colon cancer [25,26,27], lung [28], breast [24], pancreatic [17], hepatocellular carcinoma (HCC) [29], head and neck squamous cell carcinoma (HNSCC) [30], and oral cancers [31]. Several studies have reported that EpCAM plays critical roles in cancer stemness [32], cell proliferation, metabolism, angiogenesis [33], epithelial-to-mesenchymal transition (EMT) [32,34,35], metastasis [32], chemoresistance [30,36,37], and immunomodulation [38,39]. However, the role of EpCAM in cancer metastasis remains unclear. The signaling of EpCAM crosstalk with other molecules is involved in the function of CSCs in tumor development (Figure 2). EpCAM can be processed by two membranous enzymes, ADAM17 and γ-secretase [40], and is prone to cleavage during intracellular proteolysis [40,41]. The extracellular domain of EpCAM can be cut by ADAM17, leading to the shedding of EpCAM’s ectodomain (EpEX). Subsequently, the intracellular domain of EpCAM’s cytoplasmic tail (EpICD) is cleaved by γ-secretase. The released EpICD can associate with transcription factor and other cofactors, including four and a half LIM domain protein 2 (FHL2), lymphoid enhancer factor 1 (LEF1), and β-catenin [42]. The EpICD complex is translocated to the nucleus and regulates the transcription of CSC reprogramming genes, including *OCT4*, *SOX2*, *c-MYC*, and *NANOG*, which are key regulators of self-renewal [43,44] and maintenance of the oral CSCs’ population in the undifferentiated state [45,46,47]. We recently reported that increased EpEX release enhanced EpICD production. EpICD associates with β-catenin and cofactor FHL2 (four and a half LIM domain protein 2) to form a transcriptome complex that translocates into the nucleus to modulate the transcriptional activity of target genes, such as *c-Myc* and promote tumor progression [48]. Thus, precise EpCAM signaling may present an accurate relationship between EpCAM and cancer cells.

In addition to EpCAM, ADAM17 and γ-secretase also activate EGFR and other receptor tyrosine kinases (RTKs), contributing to OSCC progression [49,50]. ADAM17 expression influences the migration, adhesion, and proliferation of OSCC cells, and these cellular behaviors may be induced by EGFR/ERK1/2 signaling [49]. ADAM17-mediated EGFR activation regulates the cleavage and endocytic trafficking of the desmosomal cadherin desmoglein 2, thereby reducing cell adhesion [51]. γ-secretase activates Notch signaling, promoting the proliferation and invasion of OSCC cells, and these effects can be attenuated by a γ-secretase inhibitor [52]. A high level of MET RTK is associated with poor prognosis in HNSCC [53]. This RTK can also be cleaved by ADAM17 and γ-secretase, and the resulting MET receptor fragment, which lacks the ectodomain, contributes to cell invasion through the MAPK and PI3K/AKT signaling pathways [53,54]. Additionally, the intracellular domain fragments of ErbB4, cleaved by γ-secretase, activate other receptors, such as the NMDA receptor, facilitating cancer development through various transcription factors, including STAT5, hypoxia-inducible factor 1 alpha, and YAP [50,55,56]. Taken together, EpCAM cleavage by ADAM17 and γ-secretase promotes cancer stem cell renewal via EpICD-mediated transcription. In contrast, EGFR/RTK activation enhances tumor invasion, proliferation, and adhesion loss through ERK1/2, MAPK, and PI3K/AKT signaling. While EpCAM regulates stemness, EGFR/RTK signaling drives broader oncogenic processes, increasing malignancy and metastatic potential.

## 3. Crosstalk Between EpCAM and Other Signaling Pathways Regulating Oral CSC

In activated CSCs, several signaling pathways, including Wnt/β-catenin, as well as transforming growth factor beta 1 (TGF-β), Hedgehog (Hh), Notch, Yes-associated protein (YAP), Nuclear Factor kappa B (NF-κB), JAK/STAT3, and EGF [57], regulate cell proliferation, differentiation, and self-renewal. Some of these pathways have been reported to crosstalk with EpCAM during oral cancer progression. For example, in OSCC cells, soluble EpEX activates the EGFR-ERK signaling pathway and promotes the nuclear translocation of EpICDs, contributing to cetuximab resistance [42]. Additionally, EpCAM exerts its biological functions via Wnt/β-catenin signaling in CSCs. Yamashita et al. reported that EpCAM^+^ HCC cells exhibit high invasiveness and tumorigenicity by activating Wnt/β-catenin signaling, highlighting the critical role of EpCAM in maintaining hepatic CSCs [58]. The EpCAM gene is more highly expressed in the CSC-like side population (SP) of SAS oral cancer cells than in non-SP cells. Treatment with Honokiol, an active compound from *Magnolia officinalis*, eliminates CSC-like SP cells in SAS oral cancer by inhibiting the Wnt/β-catenin signaling pathway [59]. Additionally, in the CSC subpopulation of OSCC, CD44^+^ EpCAM^high^ cells represent proliferative epithelial CSCs with resistance to chemotherapy in oral CSCs [31,60]. However, in oral CSCs, the pathways that crosstalk with the EpCAM signaling pathway, along with their roles in self-renewal, stemness, differentiation, and other characteristics, remain unclear and require further investigation.

## 4. Risks and Causes of Oral Cancer: Genetic Mutations, Epigenetic Changes, and Post-Translational Modifications of EpCAM

Risk factors that predispose individuals to oral cancer have been identified in epidemiological (epidemiology) studies, including tobacco use [61], chewing betel, paan, and Areca [62,63] and excessive alcohol consumption [64], viral infection, poor oral hygiene, irritation caused by ill-fitting dentures and other rough surfaces on the teeth, poor nutrition [65], chronic infections caused by fungi or bacteria [66,67], and infection with oncogenic viruses, such as Human Papillomavirus (HPV) [2,68]. Additionally, clinical observations indicate that the infection of oral epithelial stem cells by high-risk human papillomavirus (HPV) types is associated with early lymphatic metastasis in HPV-related squamous cell carcinoma [69,70]. In addition, oral cancer is driven by complex interactions between environmental factors, genetic mutations, and epigenetic alterations [71].

The human EPCAM gene (*EPCAM*), also known as *TACSTD1* and located on chromosome 2P21, is comprised of nine exons [72]. Genetic mutations in *EpCAM* have been described to be responsible for congenital tufting enteropathy (CTE), intractable diarrhea in infants [73], and Lynch syndrome (also known as hereditary non-polyposis colorectal cancer or HNPCC) [74,75]. EpCAM-related Lynch syndrome is caused by deletions at the 3′-end of the *EPCAM* (*TACSTD1*) gene, resulting in promoter hypermethylation of the *MSH2* gene [74]. Oral cancers are typically associated with at least three types of genetic mutations: (1) point mutations in proto-oncogenes, (2) gene amplification, and (3) chromosomal translocation [76]. These mutations lead to the activation of oncogenes, such as epidermal growth factor receptor (*EGFR*), *BCL*, *c-MYC*, and *int-2* [77,78].

While some aspects of cancer stem cells (CSCs) differ from those of embryonic stem cells (ESCs), they also share several common properties. ESC signatures have been reported to maintain self-renewal and drive cellular reprogramming into the pluripotent state in normal somatic cells, as well as in malignant transformed cells. The exogenous induction of ESC stemness genes promotes dysplastic growth in adult epithelial tissues [79,80]. These findings highlight a potential link between ESC/stemness signature-mediated reprogramming and tumor transformation. According to recent studies, metastasis is considered a key factor for poor prognosis [81,82,83,84,85]. Additionally, EpCAM plays a crucial role in maintaining ESCs by regulating key factors, such as *c-MYC*, *OCT-4*, *NANOG*, *SOX2*, and *KLF4*, which are influenced by epigenetic alterations [45]. Epigenetic alterations include DNA methylation and histone modifications, which regulate gene expression and genome function. Promoter hypomethylation of EpCAM leads to its overexpression in various tumors, such as lung cancer [86,87], endometrial cancer [88], breast cancer [89], and ovarian cancer [90], while emphasizing its relevance in OSCC [91]. In OSCC, EpCAM expression increases during cancer development and is linked to promoter methylation; however, it does not significantly correlate with the overexpression of DNA methyltransferase-1 in OSCC tumors [91].

EpCAM has three extracellular N-glycosylation sites [92], which are crucial for maintaining its stability, expression levels, and half-life in the plasma membrane [92]. In breast cancer, N-glycosylation mutations of EpCAM reduce N-linked glycosylation, affecting the EMT [35] and cellular apoptosis by regulating the expression of both anti-apoptotic protein Bcl-2 and the pro-apoptotic proteins Bax and Caspase 3 [93], as well as cell adhesion FAK/Akt/Gsk-3β/β-catenin signaling pathway [94]. However, EpCAM is hyperglycosylated in head and neck cancer tissues [95]. Furthermore, the maintenance of CSCs has been shown to be influenced by various factors, including DNA mutations, epigenetic alterations, and genomic changes, such as chromosomal amplifications, deletions, and rearrangements, as well as interactions within the tumor microenvironment [96]. However, the role of the genetic and epigenetic modifications of *EPCAM* in regulating the characteristics of oral CSCs remains poorly understood and requires further investigation.

## 5. Role of CSCs and EpCAM Expression Within Tumor Microenvironment

CSCs residing in niches maintain self-renewal, enhance stemness, induce angiogenesis, and avoid immunosurveillance via crosstalk with immune and other stromal cells, as well as secreted factors in the tumor microenvironment [97]. These interactions are mediated through the release or encapsulation of cytokines (e.g., interleukins and TGF-β) [98,99], matrix metalloproteinases (MMPs) [100], and vascular endothelial growth factor (VEGF) [101], as well as RNA [102], DNA, lipid, and protein in extracellular vesicles (EVs), also known as exosomes. Exosomes secreted by OSCC CSCs drive M2 TAM polarization by transferring *lncRNA UCA1*, which targets the LAMC2-mediated PI3K/AKT signaling pathway while suppressing CD4^+^ T-cell proliferation and interferon-gamma (IFN-γ) production [103]. Additionally, exosomal EpCAM expression is elevated in prostate cancer patients compared to that in healthy controls [104] and has been identified as a biomarker in blood samples from pancreatic and breast cancer patients [105], where it also serves as a therapeutic target. Bi-specific antibodies targeting both EpCAM and CD73 can selectively target EpCAM^+^ carcinoma-derived EVs secreted from various cancer cells and inhibit CD73 EV-mediated immune suppression compared to CD73-targeting alone [106].

## 6. Role of EpCAM in CSC Metabolism

**Figure 3:**
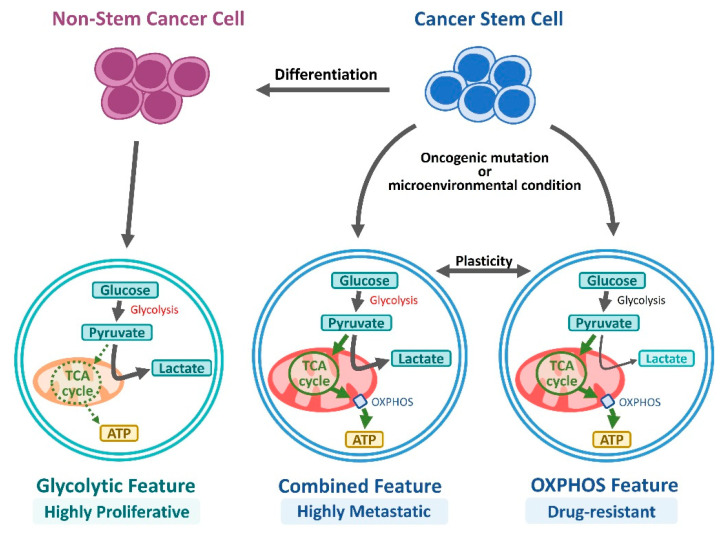
Differences in glucose utilization between CSCs and non-stem tumor cells underscore their distinct metabolic profiles. The differences in glucose utilization between CSCs and non-stem tumor cells highlight their distinct metabolic profiles. In non-stem tumor cells, metabolic reprogramming favors aerobic glycolysis, where pyruvate is predominantly converted to lactate for ATP generation. Only a minor portion of pyruvate enters the TCA cycle to support biosynthetic processes, resulting in reduced mitochondrial respiration but enhanced cell proliferation. CSCs, however, display distinct metabolic characteristics, primarily utilizing OXPHOS or employing a hybrid metabolic state that combines both OXPHOS and high glycolytic activity. This metabolic profile is dynamically regulated by various factors including oncogenic signaling, oxygen levels, and nutrient availability.

Cancer cells rely on less efficient processes and use less efficient glycolysis for the production of ATP and building essential blocks for biosynthesis (e.g., nucleotides, amino acids, and lipids) required for rapid cancer cell proliferation, providing cancer cells with a growth advantage called “The Warburg effect” [107]. As mentioned above, CSCs evolve through genetic and epigenetic alterations, as well as interactions with their niche, resulting in the emergence of diverse CSC subclones. CSCs exhibit metabolic plasticity, relying on either oxidative phosphorylation (OXPHOS), which involves mitochondrial respiration to generate ATP, or glycolysis, similar to the “Warburg effect,” depending on the oncogenic background and microenvironmental conditions, such as hypoxia or nutrient availability, with mitochondria playing a critical role in maintaining stemness, migration, and therapy resistance [108,109] (Figure 3). Oral CSCs predominantly rely on glycolysis over the oxidative phosphorylation of OXPHOS, which is a metabolic trait observed in nasopharyngeal cancer [110,111]. Metabolic reprogramming in both basal-like breast cancer CSCs and glioma CSCs involves switching from OXPHOS to aerobic glycolysis, which is crucial for maintaining CSC function by reducing ROS levels [112,113]. Additionally, in brain tumor CSCs, glucose induces the expression of key metabolic genes, including *c-MYC*, *GLUT1*, *HK-1*, *HK-2*, and *PDK-1*, which regulate glucose metabolism and activate the Akt signaling pathway [113]. In a metabolomic study of CSCs in OSCC multicellular tumor spheroids (MCTSs), CSCs were found to depend primarily on glycolysis over oxidative phosphorylation, exhibited decreased fatty acid oxidation, and showed lower metabolic activity than differentiated cancer cells, which may underlie their resistance to metabolic therapies targeting highly proliferative tumors [114]. However, the metabolic program and the underlying mechanisms of action of oral CSCs remain unclear.

## 7. EpCAM as Biomarker for Oral Cancer Diagnosis and Targeting Therapy

EpCAM^+^ circulating tumor cells (CTCs) serve as biomarkers of disease progression and metastatic risk in OSCC [115]. Circulating tumor cells (CTCs) are the seeds of metastasis [83,116,117,118]. CTCs are shed by primary tumors into the bloodstream, traveling through the vasculature via the circulation, before being deposited at distant sites and maintaining cell proliferation, triggering a cascade that is responsible for oral cancer-related deaths [119]. Unfortunately, at present, the mechanism underlying the ability of CTCs to escape and survive shear stress and the immune response in the bloodstream, as well as the locations at which they ultimately deposit, remains poorly understood. The detection of CTCs has prognostic and therapeutic implications, especially for understanding metastatic potential, disease progression, and effectiveness of treatment, as well as for providing real-time information on the disease status of patients [120]. The detection of CTCs in patients with oral squamous cell carcinoma could help predict recurrence with higher sensitivity than conventional staging [120]. Technological advances in the detection of CTCs and their bio-molecular characterization offer new perspectives for the identification of potential targets for tailor-made therapies. Hence, the early detection of tumor cell dissemination combined with an understanding of the underlying mechanisms is crucial for predicting prognosis, relapse, and survival. Furthermore, the current Food and Drug Administration (FDA)-approved CTC assay, the CellSearch™ System (Veridex LLC, Raritan, NJ, USA; CellSearch) [121,122,123], overlooks CSC phenotypes, crucial for tumor progression and therapy resistance, prompting the integration of CTC and CSC markers for improved prognostic accuracy [124].

## 8. EpCAM-Targeting Immunotherapies

Inhibitors targeting specific molecules involved in tumor progression or their downstream signaling pathways have been used to improve disease prognosis [125,126]. Various EpCAM-targeted immunotherapies that demonstrate promising anticancer activity against oral cancer cells and CSCs, including targeted antibodies, chimeric antigen receptor (CAR) T or NK cells, have been developed.

First, there is a human monoclonal antibody against EpCAM, adecatumumab, which has been evaluated in clinical studies for its potential to inhibit tumor growth and metastasis in breast cancers [127]. Anti-EpCAM monoclonal antibody (EpMab-16) demonstrates in vivo anti-OSCC activity via the induction of antibody-dependent cellular cytotoxicity (ADCC) and complement-dependent cytotoxicity (CDC) [128]. Second, low doses of the EpCAM/CD3-bispecific T-cell engager (BiTE) antibody MT110 (solitomab) effectively engage cytotoxic human T cells and demonstrate the potential to target highly tumorigenic pancreatic CSCs, both in vitro and in vivo [129]. Similarly, BiTE solitomab demonstrates anti-tumor activity against primary uterine and ovarian carcinosarcoma cells [130]; however, it failed to surpass clinical trials due to gastrointestinal toxicity [131]. EpCAM-targeted antibodies exhibit anticancer activity against tumors or CSCs not only by directly targeting cancer cells but also by regulating the expression of immune checkpoint inhibitors. For example, EpCAM antibodies (EpAb2-6) effectively downregulate PD-L1 levels, enhance CD8^+^ T-cell cytotoxic activity, and boost the therapeutic efficacy of atezolizumab, an anti-PD-L1 antibody, in vivo [38].

IL-15, a cytokine that activates NK cells [132], promotes the expansion of CAR-NK cells in vivo and enhances their cytotoxicity against EpCAM^+^ breast CSCs [133]. In addition, EpCAM-specific CAR-NK-92 cells have been shown to exhibit a strong potential to kill CRC cells, with their effects being further enhanced in combination with regorafenib, a potent multikinase inhibitor.

EpCAM CAR-T cells have been developed that exhibit effective killing abilities against various tumors, such as AML [134], gastric [135], colon/lung/pancreatic [136], and prostate cancer [137]. EpCAM CAR-T cells effectively induce apoptosis in colon cancer cells and enhance the secretion of cytokines IL-2, IFN-γ, and IL-6, which play antitumor roles in immunotherapy by modulating immune responses [138,139,140]. The action of EpCAM CAR-T cells against solid tumors, as well as their safety, was evaluated using an EpCAM-humanized mouse model, with clinical trials of autologous EpCAM CAR-T cell therapy for solid tumors demonstrating both safety and efficacy [141].

CSC-derived peptides, including EpCAM peptides used as antigen sources for dendritic cell (DC) vaccination, induce EpCAM peptide-specific cytotoxic T lymphocytes (CTLs) with potent cytotoxic activity against EpCAM-positive HCC cells [142]. Additionally, EpCAM, a common tumor-associated antigen (TAA) targeted in colon cancer, induces IL-4-dominated T helper (Th)2 responses during Th-cell priming, even under Th1-inducing conditions, promoting tumor growth and undermining the therapeutic efficacy of tumor vaccines aimed at inducing interferon-γ (IFN-γ)-producing CD4^+^ Th1 cells [143]. However, the efficacy of EpCAM CAR-T cells, NK cells, and vaccines against oral cancer or CSCs have yet to be characterized.

The RNA EpCAM-aptamer-based delivery system (Apt-DOX) targets colon CSCs, enhancing DOX retention in the nuclei, significantly improving CSC sensitivity to DOX, overcoming chemoresistance, and eliminating CSCs both in vitro and in vivo [144]. The novel synthesis of doxorubicin hydrochloride (DOX·HCl) and siRNA-loaded polymer vesicles labeled with anti-EpCAM antibody demonstrated effective liver CSC killing and tumor growth inhibition with reduced toxicity to normal cells in vitro [145].

However, the heterogeneous expression of EpCAM in carcinomas, along with its presence in normal epithelial tissues, raises concerns about potential off-target effects and toxicity [146]. Therefore, strategies to modulate EpCAM within the tumor microenvironment must carefully balance therapeutic efficacy with safety. Further research is warranted to elucidate the complex role of EpCAM in tumor biology and to develop targeted therapies that effectively disrupt its function within the TME while minimizing adverse effects on normal tissues (Table 1).

## 9. Limitations of EpCAM-Based Therapies

EpCAM-based therapies face several challenges, primarily due to the heterogeneity and plasticity of cancer cells. One major limitation is the variable EpCAM expression in CTC. Many CTCs undergo epithelial-to-mesenchymal transition (EMT), leading to the downregulation of EpCAM and a mesenchymal phenotype [121,147,148]. Additionally, other EMT+ cells, circulating tumor stem cells (CTSCs), downregulate epithelial markers such as EpCAM while upregulating mesenchymal markers, and epithelial tumor cells, EMT^+^ tumor cells, and CTSCs coexist simultaneously in peripheral blood [149,150,151,152,153,154]. This issue is particularly evident in cancers such as breast cancer [121]. Also, during tumor progression, dynamic changes in EpCAM expression occur, prompting the use of enrichment systems that enable the simultaneous investigation of both EpCAM^+^ and EpCAM^−^ CTCs to gain a more comprehensive understanding of EpCAM’s role in tumor progression [155]. Accordingly, those systems reduce the effectiveness of EpCAM-targeted detection and therapies.

To address these limitations, EpCAM-independent CTC detection methods are being explored, including size-based filtration ISET [156,157], a microfluidic systems Parsortix^®^ PC1 [158], and multiplex approaches that combine EpCAM with other epithelial (e.g., HER2, HER3, EGFR, and MUC1) [159,160,161,162], or mesenchymal markers (e.g., vimentin, N-cadherin) [163,164,165,166]. Additionally, immunomagnetic enrichment techniques (such as CellSearch [121,122,123,167,168,169,170,171], MACS [172,173], MagSweeper [174,175], Strep-tag [176,177], IMS [178]) targeting alternative surface proteins are under investigation. Accordingly, more comprehensive targeting strategies are needed, those integrating EpCAM-based approaches with alternative technologies to enhance the efficacy of cancer diagnosis and therapy.

## 10. Summary

This review focuses on the critical role of the Epithelial Cell Adhesion Molecule (EpCAM) in oral squamous cell carcinoma (OSCC), emphasizing its contributions to cancer stem cell (CSC) biology, tumor progression, and therapeutic resistance. EpCAM is a transmembrane glycoprotein that functions in cell adhesion and signaling. Its overexpression is associated with a poor prognosis and is a hallmark of CSCs in various cancers, including OSCC. EpCAM mediates tumorigenicity by promoting the EMT, metastasis, and therapy resistance through pathways including Wnt/β-catenin and EGFR-ERK, among others. Additionally, EpCAM signaling interacts with metabolic reprogramming to facilitate CSC adaptability and survival in adverse tumor microenvironments.

Taken together, this review explored the potential of EpCAM as a biomarker for early cancer detection, as well as a target for innovative therapies, including CAR-T cells, monoclonal antibodies, and EpCAM-labeled drug delivery systems. This discussion highlights the limitations of current therapeutic strategies and the need for the more precise targeting of EpCAM-mediated pathways.

## 11. Conclusions

EpCAM plays a pivotal role in the progression, metastasis, and therapeutic resistance of OSCC by regulating the CSC properties and tumor microenvironment interactions. Its dual functionality as a biomarker and therapeutic target makes it a promising candidate for advancing personalized cancer treatment. Recent advancements in artificial intelligence (AI) have shown promise in enhancing the detection of EpCAM-expressing CTCs. A convolutional neural network-based image processing algorithm has demonstrated higher accuracy and reduced analysis time compared to manual methods, suggesting its potential applicability in clinical settings [179]. As mentioned above, there are several limitations identified, including the fact that EpCAM is also expressed on normal epithelial cells, raising concerns about potential off-target effects and toxicity [146]; the dynamic expression of EpCAM during tumor progression might affect therapeutic efficacy [121,147,148]; and its expression differs among various tumor types, as well as between primary and metastatic tumors [86,180]. Future research should aim to overcome these challenges by developing strategies that enhance the specificity and efficacy of EpCAM-targeted therapies while minimizing adverse effects on normal tissues. Integrating AI technologies for precise detection and monitoring of EpCAM expression, coupled with a deeper understanding of EpCAM’s role in tumor biology, could pave the way for more effective and safer therapeutic approaches. However, further studies are needed to elucidate the precise mechanisms underlying EpCAM signaling and address the challenges of targeting CSCs to improve the efficacy and safety of EpCAM-based therapies.

## Figures and Tables

**Table 1:** Comparative table of EpCAM-targeted therapeutic approaches.

Therapy Type	Agent	Mechanism of Action	Cancer Types Investigated	Clinical Efficacy	Side Effects/Limitations	References
Monoclonal Antibody	Adecatumumab	Human monoclonal antibody against EpCAM; inhibits tumor growth and metastasis	Breast cancer	Evaluated in clinical trials; reduced tumor proliferation	Limited efficacy in solid tumors, off-target toxicity	[127]
Monoclonal Antibody	EpMab-16	Induces antibody-dependent cellular cytotoxicity (ADCC) and complement-dependent cytotoxicity (CDC)	OSCC	In vivo anti-OSCC activity	Requires further validation in clinical trials	[128]
BiTE	Solitumab (MT110)	EpCAM/CD3-targeting to engage cytotoxic T cells	Pancreatic, uterine, ovarian carcinosarcoma	Demonstrated anti-tumor activity in vitro and in vivo	Failed clinical trials due to gastrointestinal toxicity	[129,130,131]
Immune Checkpoint Modulation	EpAb2-6 + Atezolizumab	Downregulates PD-L1 and enhances CD8+ T-cell cytotoxicity	Solid tumors	Increased efficacy of anti-PD-L1 therapy	Requires further clinical evaluation	[38]
CAR-NK Cells	IL-15-stimulated CAR-NK	Enhances NK cell cytotoxicity against EpCAM^+^ CSCs	Breast, colorectal cancer	Synergistic effect with regorafenib; strong in vitro killing	NK persistence and in vivo efficacy require further study	[132,133]
CAR-T Cells	EpCAM CAR-T	Targets EpCAM-expressing tumor cells, induces cytokine secretion	AML, gastric, colon, lung, pancreatic, prostate cancer	Effective tumor killing, apoptosis induction, cytokine secretion	Potential toxicity; heterogeneity in EpCAM expression	[134,135,136,137,138,139,140,141]
DC Vaccination	EpCAM Peptides	Induces EpCAM peptide-specific CTLs with cytotoxic activity	Hepatocellular carcinoma	Effective cytotoxic response in vitro	Potential immunosuppressive effects	[142]
RNA Aptamer-Based Therapy	Apt-DOX	EpCAM-aptamer targets colon CSCs, enhances DOX retention	Colon cancer	Overcomes chemoresistance, eliminates CSCs in vitro and in vivo	Requires further preclinical/clinical validation	[143]
siRNA Delivery System	DOX·HCl-siRNA Vesicles	EpCAM-targeted polymer vesicles for CSC killing	Liver cancer	Reduced toxicity to normal cells, strong anti-CSC effect	Stability and in vivo delivery need optimization	[144,145]

## Data Availability

Not applicable.
